# Potential Protein Phosphatase 2A Agents from Traditional Chinese Medicine against Cancer

**DOI:** 10.1155/2014/436863

**Published:** 2014-04-29

**Authors:** Kuan-Chung Chen, Hsin-Yi Chen, Calvin Yu-Chian Chen

**Affiliations:** ^1^School of Pharmacy, China Medical University, Taichung 40402, Taiwan; ^2^Department of Biomedical Informatics, Asia University, Taichung 41354, Taiwan; ^3^School of Medicine, College of Medicine, China Medical University, Taichung 40402, Taiwan; ^4^Computational and Systems Biology, Massachusetts Institute of Technology, Cambridge, MA 02139, USA

## Abstract

Protein phosphatase 2A (PP2A) is an important phosphatase which regulates various cellular processes, such as protein synthesis, cell growth, cellular signaling, apoptosis, metabolism, and stress responses. It is a holoenzyme composed of the structural A and catalytic C subunits and a regulatory B subunit. As an environmental toxin, okadaic acid, is a tumor promoter and binds to PP2A catalytic C subunit and the cancer-associated mutations in PP2A structural A subunit in human tumor tissue; PP2A may have tumor-suppressing function. It is a potential drug target in the treatment of cancer. In this study, we screen the TCM compounds in TCM Database@Taiwan to investigate the potent lead compounds as PP2A agent. The results of docking simulation are optimized under dynamic conditions by MD simulations after virtual screening to validate the stability of H-bonds between PP2A-**α** protein and each ligand. The top TCM candidates, trichosanatine and squamosamide, have potential binding affinities and interactions with key residues Arg89 and Arg214 in the docking simulation. In addition, these interactions were stable under dynamic conditions. Hence, we propose the TCM compounds, trichosanatine and squamosamide, as potential candidates as lead compounds for further study in drug development process with the PP2A-**α** protein.

## 1. Introduction

Protein phosphatase 2A (PP2A) is an important phosphatase which consists of a holoenzyme composed of the structural A and catalytic C subunits and a regulatory B subunit [1–3]. As each of these subunits exists many different isoforms, the holoenzymes of PP2A, can form various distinct trimeric ABC complexes. This enzyme can regulate various cellular processes, such as protein synthesis, cell growth, cellular signaling, apoptosis, metabolism, and stress responses [4, 5]. Many researches indicate the cancer-associated mutations in PP2A structural A subunit in human tumor tissue [6–8]. As a research in 1988 determined that an environmental toxin, okadaic acid, is a tumor promoter and binds to PP2A catalytic C subunit [9], PP2A may have tumor-suppressing function. As PP2A has tumor-suppressing function, it is a potential drug target in the treatment of cancer [10, 11].

Nowadays, the researchers have determined more and more distinct mechanisms of diseases [12–18]. According to these mechanisms, the researchers can identify the potential target protein for drug design against each disease [19–22]. The compounds extracted from traditional Chinese medicine (TCM) have been indicated in many* in silico* researches as potential lead compounds for the treatment of many different diseases, including tumours [23–26], diabetes [27], inflammation [28], influenza [29], metabolic syndrome [30], stroke [31–33], viral infection [34], and some other diseases [35, 36]. In this study, we aim to improve drug development of TCM compounds by investigating the potent lead compounds as PP2A agent from the TCM compounds in TCM Database@Taiwan [37]. As the disordered amino acids in the protein may cause the side effect and reduce the possibility of ligand binding to target protein [38, 39], we have predicted the disordered residues in sequence of PP2A-*α* protein before virtual screening. After virtual screening of the TCM compounds, the results of docking simulation are optimized under dynamic conditions by MD simulations to validate the stability of H-bonds between PP2A-*α* protein and each ligand.

## 2. Materials and Methods

### 2.1. Data Collection

The X-ray crystallography structure of the human serine/threonine-protein phosphatase 2A (PP2A) catalytic subunit alpha isoform was obtained from RCSB Protein Data Bank with PDB ID: 3FGA [40]. We employed PONDR-Fit [41] protocol to predict the disordered residues in sequence of PP2A-*α* protein from Swiss-Prot (UniProtKB: P67775). For preparation, the protein was protonated with Chemistry at HARvard Macromolecular Mechanics (CHARMM) force field [42], and the crystal water was removed using Prepare Protein module in Discovery Studio 2.5 (DS2.5). The volume of the cocrystallized PP2A inhibitor, microcysteine, was employed to define the binding site for virtual screening. TCM compounds from TCM Database@Taiwan [37] were protonated using Prepare Ligand module in DS2.5 and filtered by Lipinski's Rule of Five [43] before virtual screening.

### 2.2. Docking Simulation

For virtual screening, the TCM compounds were docked into the binding site using a shape filter and Monte-Carlo ligand conformation generation using LigandFit protocol [44] in DS2.5. The docking poses were then optionally minimized with CHARMM force field [42] and then calculated their Dock Score energy function by the following equation:

(1) Dock  Score=−(ligand/receptor  interaction  energy   +ligand  internal  energy).

Finally, the similar poses were rejected using the clustering algorithm.

### 2.3. Molecular Dynamics (MD) Simulation

The molecular dynamics (MD) simulation for each protein-ligand complex under dynamic conditions was performed by Gromacs 4.5.5 [45]. The topology and parameters for PP2A-*α* protein with charmm27 force field and ligands were performed by the pdb2gmx protocol of Gromacs and SwissParam program [46], respectively. Gromacs performed a cubic box with edge approx 12 Å from the molecules periphery and solvated with TIP3P water model for each protein-ligand complex. The common minimization algorithm, Steepest descents [47], was employed with a maximum of 5,000 steps to remove bad van der Waals contacts. After a neutral system using 0.145 M NaCl model was created by Gromacs; the steepest descents minimization with a maximum of 5,000 steps was employed again to remove bad van der Waals contacts. For the equilibration, the Linear Constraint algorithm for all bonds was employed for the position-restrained molecular dynamics with NVT equilibration, Berendsen weak thermal coupling method, and Particle Mesh Ewald method. A total of 5000 ps production simulation was then performed with time step in unit of 2 fs under Particle Mesh Ewald (PME) option and NPT ensembles. The 5000 ps of MD trajectories was then analyzed using a series of protocols in Gromacs.

## 3. Results and Discussion

### 3.1. Disordered Protein Prediction

**Figure 1:**
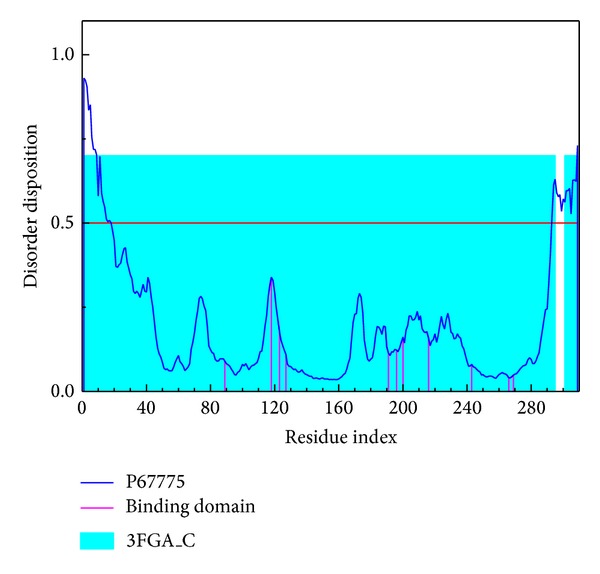
Disordered disposition predicted by PONDR-Fit.

The result of the disordered residues predicted by PONDR-Fit with the sequence of PP2A-*α* protein from Swiss-Prot (UniProtKB: P67775) is illustrated in Figure 1. For PP2A-*α* protein, Figure 1 indicates that the structure of binding domain is stable as the major residues of binding domain do not lie in the disordered region.

### 3.2. Docking Simulation

**Figure 2:**
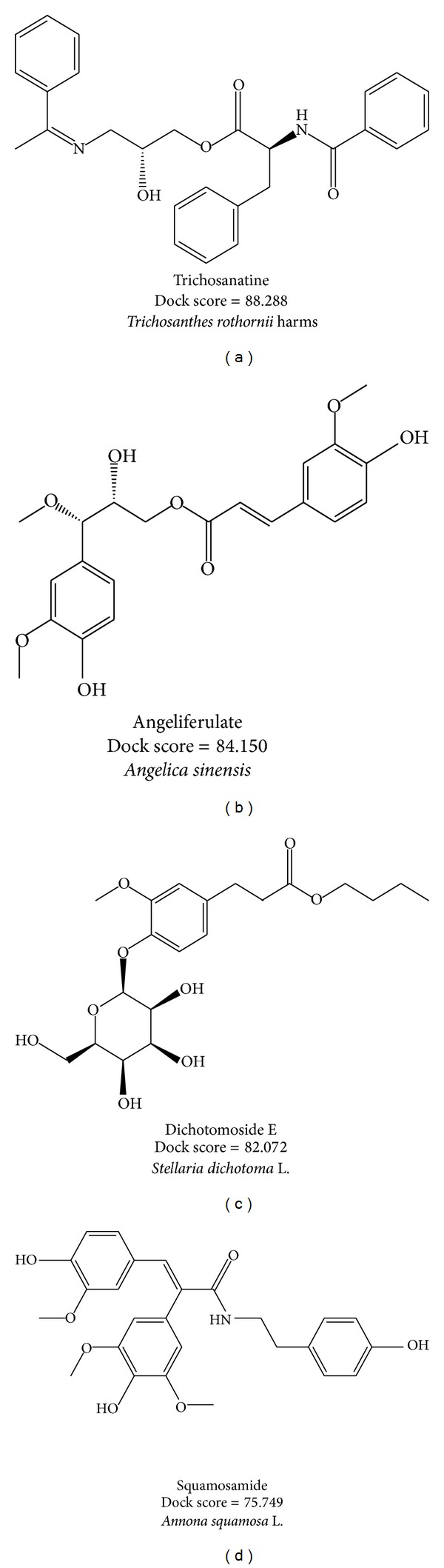
Chemical scaffold of top four TCM candidates with their scoring function and sources.

**Figure 3:**
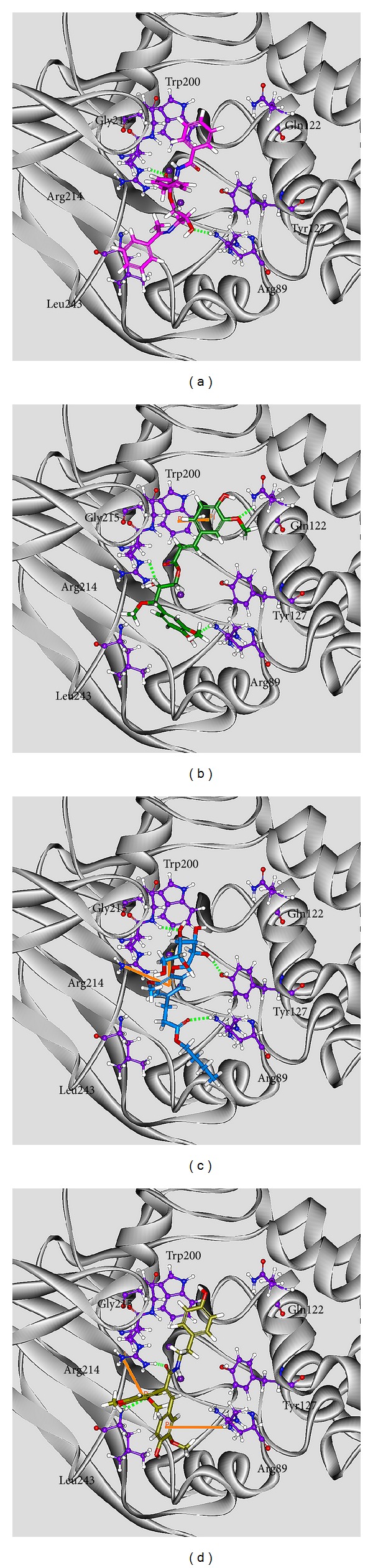
Docking pose of PP2A protein complexes with (a) trichosanatine, (b) angeliferulate, (c) dichotomoside E, and (d) squamosamide.

For virtual screening, Dock Score energy function is used to rank the top potential TCM compounds; the chemical scaffold of top four TCM candidates with high binding affinity is displayed in Figure 2 with its scoring function and sources. The top four TCM compounds, trichosanatine, angeliferulate, dichotomoside E, and squamosamide, were extracted from* Trichosanthes rosthornii* Harms,* Angelica sinensis*,* Stellaria dichotoma* L., and* Annona squamosa* L., respectively. After the virtual screening, the docking poses of top four TCM compounds in the binding domain of PP2A-*α* are displayed in Figure 3. All the top four TCM compounds have interactions with key residues Arg89 and Arg214. Trichosanatine exists hydrogen bonds (H-bonds) with key residues Arg89 and Arg214. Angeliferulate has H-bonds with residues Arg89, Gln122, Arg214, and a *π* interaction with residue Trp200. Dichotomoside E forms H-bonds with residues Arg89, Tyr127, Gly215, and a *π* interaction with residue Arg214. Squamosamide has both H-bond and *π* interaction with residue Arg214. In addition, there exist H-bonds with residue Leu243 and a *π* interaction with residue Arg89. Those interactions hold the top four TCM compounds in the binding domain of PP2A-*α* protein.

### 3.3. Molecular Dynamics Simulation

**Figure 4:**
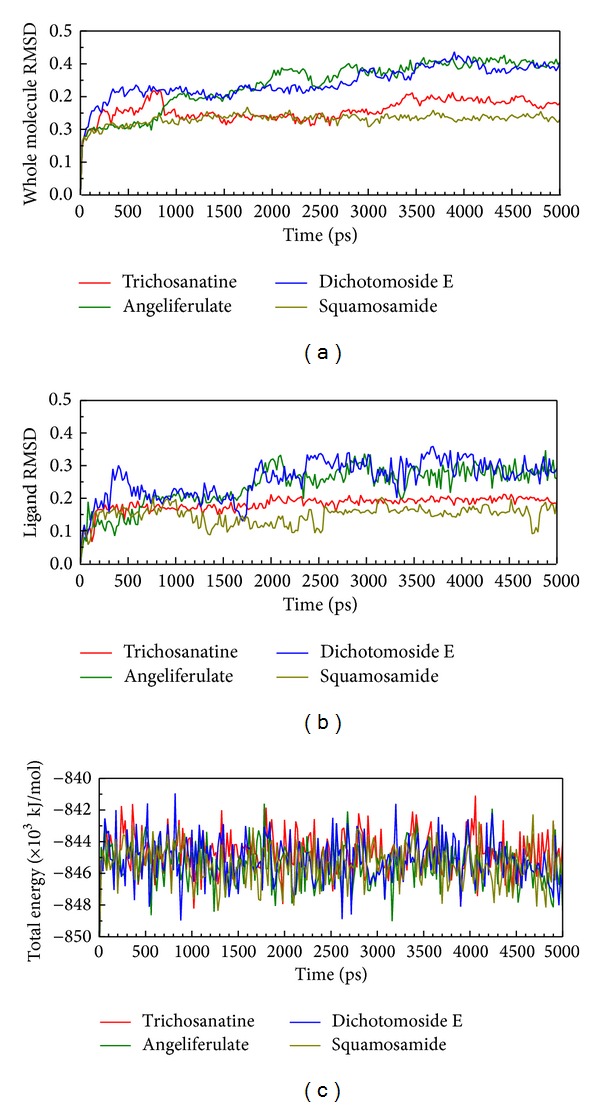
Root-mean-square deviations in units of nm and total energies over 5000 ps of MD simulation for PP2A protein complexes with trichosanatine, angeliferulate, dichotomoside E, and squamosamide.

**Figure 5:**
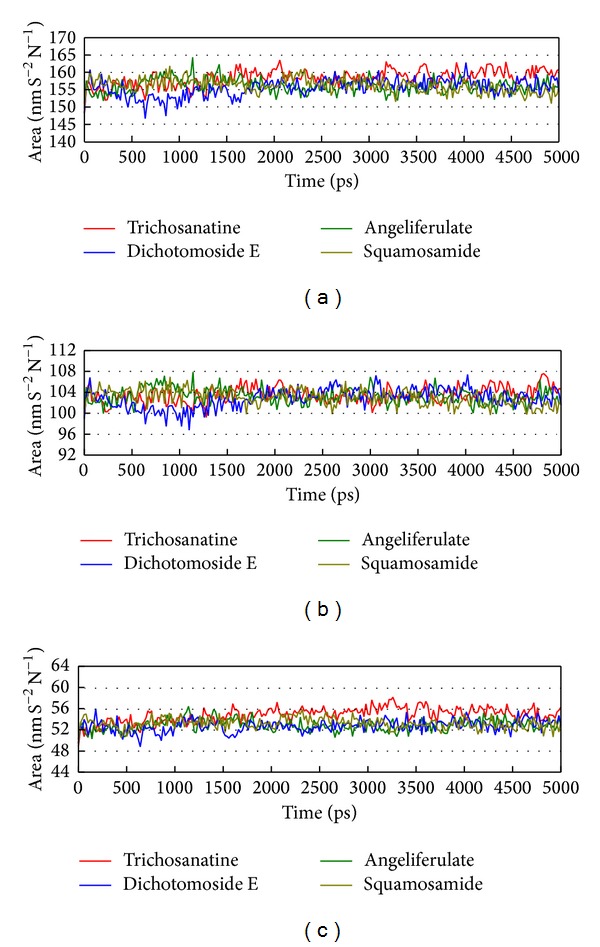
Variation of (a) total solvent accessible surface area, (b) hydrophobic surface area, and (c) hydrophilic surface area over 5000 ps of MD simulation for PP2A protein complexes with trichosanatine, angeliferulate, dichotomoside E, and squamosamide.

**Figure 6:**
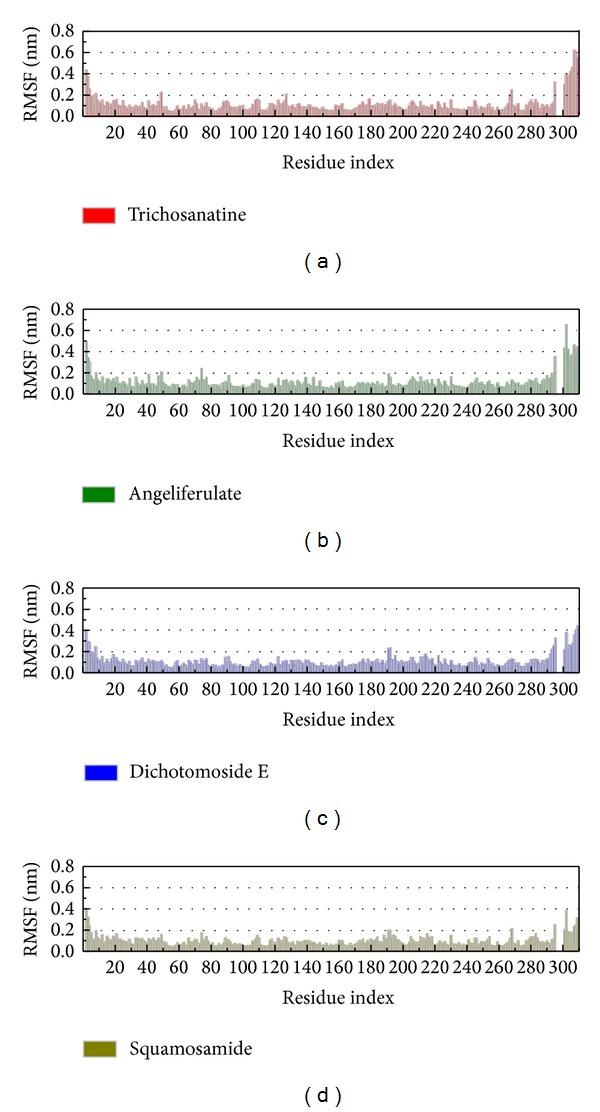
Root-mean-square fluctuation in units of nm for residues of PP2A protein complexes with trichosanatine, angeliferulate, dichotomoside E, and squamosamide during 5000 ps of MD simulation.

**Figure 7:**
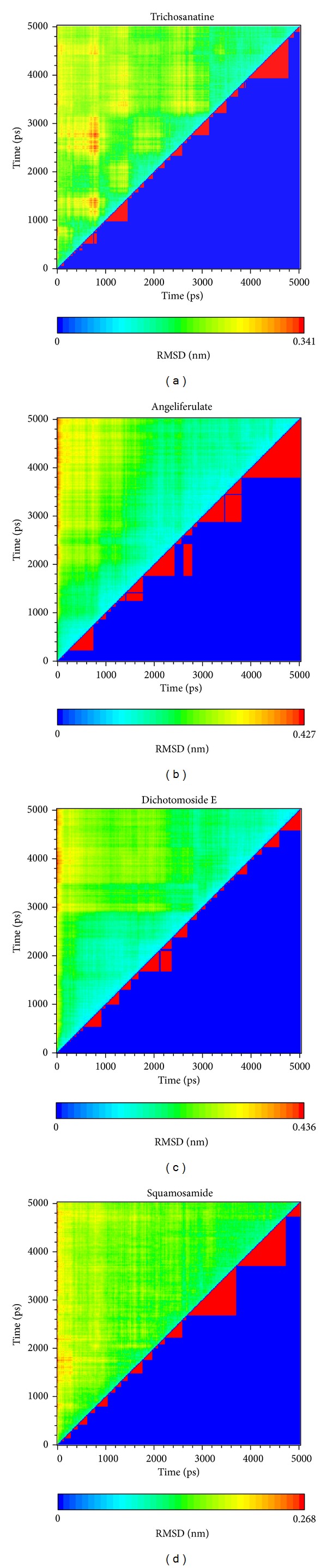
Root-mean-square deviation value (a) and graphical depiction of the clusters with cutoff 0.1 nm (d) for PP2A protein complexes with trichosanatine, angeliferulate, dichotomoside E, and squamosamide.

**Figure 8:**
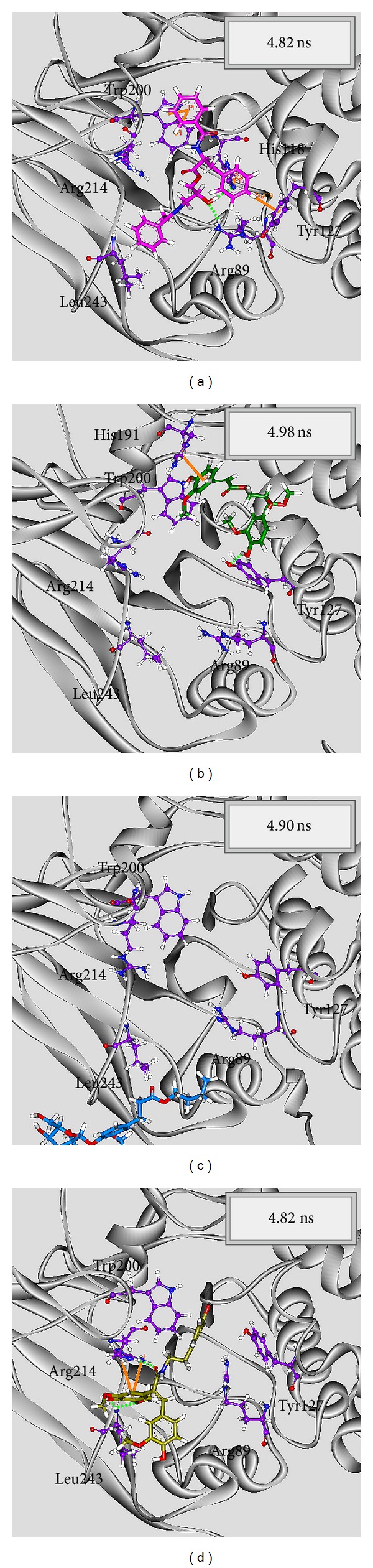
Docking poses of middle RMSD structure in the major cluster for PP2A protein complexes with trichosanatine, angeliferulate, dichotomoside E, and squamosamide.

**Figure 9:**
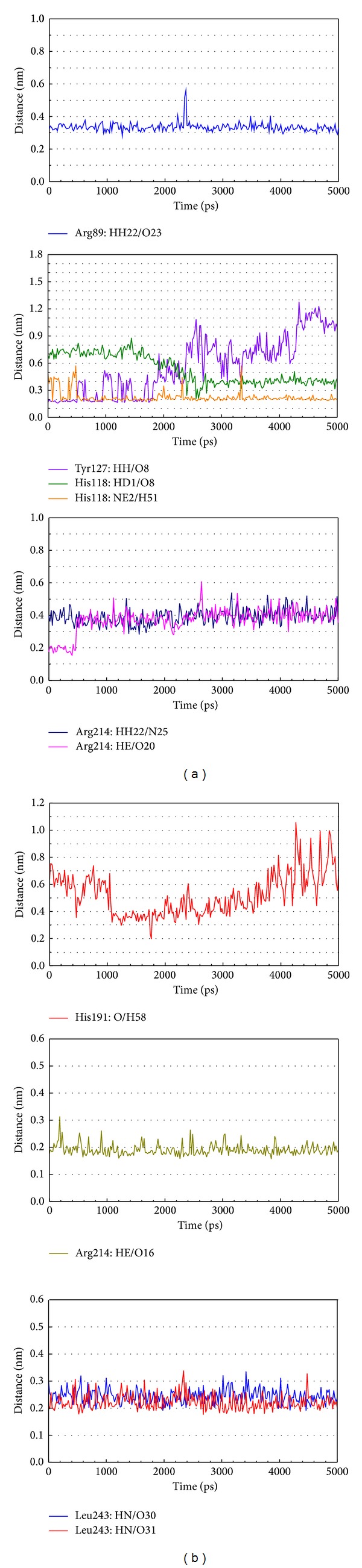
Distances of hydrogen bonds with common residues during 5000 ps of MD simulation for PP2A protein complexes with trichosanatine and squamosamide.

In LigandFit protocol, each compound was docked into binding site using a shape-based docking with rigid body of PP2A-*α* protein. The interactions between each compound and PP2A-*α* protein mention above may not be stable under dynamic conditions. We employed MD simulation for each protein-ligand complex to study the stability of interactions for each docking pose. The information of root-mean-square deviations (RMSDs) and total energies over 5000 ps of MD simulation is displayed in Figure 4. It indicates that the atomic fluctuations of protein complexes with top four TCM compounds tend to be stable after 4800 ps of MD simulation, and there is no significant variation in the total energies for each complex during MD simulation. To analyze the possible effect of each top TCM candidate for the PP2A-*α* protein, Figure 5 displays the variation of solvent accessible surface area for PP2A-*α* protein over 5000 ps of MD simulation. For top four TCM candidates, they have similar hydrophobic and hydrophilic surface areas when the MD simulation tends to be stable, which indicates that those compounds may not affect the sharpness of PP2A-*α* protein after they dock in the binding domain.  Figure 6 illustrates the root-mean-square fluctuation of each residue of PP2A-*α* protein during 5000 ps of MD simulation. It indicates that they have similar deviation for key residues in the binding domain of PP2A-*α* protein during 5000 ps of MD simulation. In Figure 7, root-mean-square deviation value for each PP2A-*α* protein complex illustrates the RMSD values between each MD trajectory of 5000 ps of MD simulation, and graphical depiction of the clusters with cutoff 0.1 nm is employed to define the middle RMSD structure in the major cluster as the representative structures for each complex after MD simulation. The docking poses of the representative structures for each protein-ligand complex are illustrated in Figure 8. For angeliferulate and dichotomoside E, the interactions between protein and ligand mention in docking simulation are not stable under dynamic conditions, which indicates that those two TCM compounds cannot binding stabilized in the binding domain of PP2A-*α* protein. For trichosanatine, the representative docking poses in 4.82 ns indicate that it has similar docking pose as mentioned in docking simulation and maintains the H-bond with key residue Arg89. In addition, it forms H-bonds and *π* interactions with residues His118, Tyr127, and Trp200 after MD simulation. The docking pose of squamosamide in 4.82 ns of MD simulation also has similar docking pose as mentioned in docking simulation and maintains the H-bond and *π* interactions with key residue Arg214 and H-bond with Leu243. To analyze the stability of these H-bonds, the occupancy of H-bonds overall 5000 ps of molecular dynamics simulation are listed in Table 1, and the variations of distance for each H-bond in the PP2A-*α* protein complexes with trichosanatine and squamosamide are illustrated in Figure 9. For trichosanatine, it has stable H-bonds with residues Arg89 and His118, and the distances with residues Arg214 are stable in 0.4 nm. For squamosamide, it has stable H-bonds with residues Arg214 and Leu243.

## 4. Conclusion

This study aims to investigate the potent TCM candidates as lead compounds of agent for PP2A-*α* protein. The top four TCM compounds have high binding affinities with PP2A-*α* protein in the docking simulation. However, the results of docking simulation are optimized under dynamic conditions by MD simulations to validate the stability of H-bonds between PP2A-*α* protein and each ligand. Although angeliferulate and dichotomoside E have potent binding affinities with PP2A-*α* protein in the docking simulation, the interactions between protein and ligand mentioned in docking simulation are not stable under dynamic conditions. For the other two top TCM candidates, trichosanatine and squamosamide, there exist stable interactions with key residues Arg89 and Arg214 under dynamic conditions. Hence, we propose the TCM compounds, trichosanatine and squamosamide, as potential candidates as lead compounds for further study in drug development process with the PP2A-*α* protein.

## Figures and Tables

**Table 1:** H-bond occupancy for key residues of PP2A protein with trichosanatine and squamosamide overall 5000 ps of molecular dynamics simulation.

Name	H-bond interaction		Occupancy
Trichosanatine	Arg89 : HH22	/O23	2%
	His118 : HD1	/O8	2%
	His118 : NE2	/H51	92%
	Tyr127 : HH	/O8	26%
	Arg214 : HE	/O20	11%
	Arg214 : HH22	/N25	1%

Squamosamide	His191 : O	/H58	1%
	Arg214 : HE	/O16	100%
	Leu243 : HN	/O30	98%
	Leu243 : HN	/O31	98%

H-bond occupancy cutoff: 0.3 nm.
